# Unlocking the Value of Patient Self-Monitoring Technologies: A Multi-Country Framework to Calibrate Payer and HTA Coverage and Reimbursement Considerations with Innovation

**DOI:** 10.36469/001c.163078

**Published:** 2026-06-16

**Authors:** James K. Karichu, Shafie Kamaruddin, Pinar Bilir, Paco Cerletti, Susan N. Chang, Sumudu Dehipawala, Conor Maher, Abbie Malyon, Matthew O’Hara

**Affiliations:** 1 Roche Molecular Systems, Pleasanton, California, USA; 2 County Durham and Darlington NHS Foundation Trust, Darlington, UK; 3 Trinity Life Sciences, Waltham, Massachusetts, USA; 4 Roche Diagnostics International AG, Basel, Switzerland; 5 Roche Diagnostics Limited, Burgess Hill, UK; 6 Trinity Life Sciences

**Keywords:** coverage, reimbursement, evidence strategy, health technology assessment, patient self-monitoring, medical devices, value framework

## Abstract

**Background:**

Patient self-monitoring (PSM) is emerging as an innovative modality for chronic disease management. While PSM technologies are established in therapeutic areas including diabetes and cardiovascular diseases, current payer and health technology assessments (HTA) do not adequately evaluate the unique facets of their value.

**Objectives:**

This study developed a multi-country, stakeholder-informed framework that considers the value drivers of PSM technologies, mapping these to payer and HTA evaluation criteria to guide evidence generation and reimbursement decision making.

**Methods:**

A sequential mixed methodology study of an artificial intelligence-enabled targeted literature review using Nested Knowledge® and qualitative primary research with 10 payer and healthcare practitioner respondents in the United States, United Kingdom, and Germany was used to inform development of the framework.

**Results:**

The framework comprises three interconnected core components: value drivers, evaluation criteria, and evidence generation considerations. The value drivers component consists of four themes: clinical, economic, humanistic, and societal. Primary research respondents weighted the respective emphasis of decision making as 60% clinical, 20% economic, 10% humanistic, and 10% societal value. Key value drivers included treatment efficacy/clinical utility, diagnostic efficacy, and healthcare resource utilization. Evaluation criteria indicate the coverage/reimbursement requirements of key payer and HTA organizations. Evidence generation considerations comprise potential study types and evidence generation activities.

**Conclusions:**

This framework provides a conceptual foundation for stakeholders including payers, HTA bodies, physicians, and developers to align upon evidence requirements for PSM technologies. It can support streamlined strategic evidence generation and consistent evaluation to improve patient access to innovative chronic disease self-monitoring technologies.

## INTRODUCTION

The escalating global prevalence of chronic diseases presents an urgent unmet need for value-driven healthcare innovation to reduce burden on healthcare systems. Over three-quarters of the US adult population live with at least 1 chronic condition, and over half manage multiple chronic conditions, including diabetes, hypertension, and arthritis.^1^ These rates are projected to nearly double by 2050, stretching the capacity of healthcare systems and prompting a shift toward innovative strategies for healthcare self-management.^2^

Patient self-monitoring (PSM), a modality by which individuals independently measure health-related data using regulated medical devices designed for use outside the clinic, represents a shift in chronic disease management by empowering individuals to actively collect health data and manage their own care. A core aspect of PSM is thus patient actionability and the extent to which they can interpret and repeatably act on the data generated. This involves active re-engagement with monitoring outputs, enabling patients to translate data insights into informed self-management decisions through an ongoing feedback loop. This actionability distinguishes PSM from other data collection methods, such as telehealth technologies and provider-centric care models, which focus on directly informing physicians of patients’ health changes (either manually or automatically). PSM, by contrast, enables patients to take direct action, for example, by self-titrating medication, implementing lifestyle modifications, or initiating other interventions.^3^

### Current Use Cases of PSM

PSM is an established modality in diabetes management, with continuous glucose monitoring (CGM) and self-monitoring of blood glucose,^4,5^ and in cardiovascular diseases via home blood pressure monitors for hypertension and international normalized ratio monitors for anticoagulation management.^6,7^ Innovation in PSM continues to occur across additional emerging therapeutic areas, such as asthma, oncology, rheumatology, and neurological conditions, where relevant value to payers/ health technology assessment (HTA) bodies is less understood.^8–11^

### Value Proposition of PSM

The clinical, economic, and humanistic impact of PSM has been demonstrated in established therapeutic areas, via improvements in clinical outcomes, reduction in healthcare resource utilization (HCRU), and enhancement of patient quality of life (QoL)^12–23^ For example, a US real-world evidence (RWE) study demonstrated that initiation of CGM was associated with significant reductions in glycated hemoglobin (HbA1c; −0.7%, *P* < .0001), as well as diabetes-related hospitalizations and emergency department visits (67% and 40% reductions, respectively; *P* < .0001).^18^ Additionally, PSM provides value by empowering patients to engage beyond medication adherence, supporting broader disease management and lifestyle modifications.^15,24^

### Disparity in PSM Coverage, Reimbursement, and Adoption

Despite the proven benefits in diabetes and cardiovascular diseases, the integration of PSM across the broader healthcare landscape remains limited and inconsistent, suggesting a need for well-structured evidence and value assessment methodology.^25,26^ Lack of a consistent and adequate value framework to guide coverage and reimbursement decisions may indeed be a key contributing factor to limited adoption of PSM technologies. For example, while CGMs have existing and well-defined reimbursement pathways in diabetes, emerging PSM technologies in oncology or rheumatology face ambiguous evidence requirements.^8,10,24^ This is compounded by a lack of healthcare professional (HCP) awareness and education on PSM effectiveness, insufficient dedicated onboarding programs leading to inefficient HCP and patient use of PSMs, unresolved medicolegal considerations (eg, data privacy, user error), and unknown sustained treatment efficacy/clinical utility benefits.^27^

The global disparity in evidence requirements may stem from the absence of a standardized framework for assessing evidence and value, even in established therapeutic areas such as diabetes. This reduces incentive for developers to innovate such technologies, limiting patient access to PSM modalities, and growing coverage and reimbursement gaps, especially as regulatory scrutiny increases.

The absence of a standardized framework can result in uncertainty among evidence generation requirements from payers and HTA bodies and inconsistent payer coverage criteria.^28^ Without clear guidance, study designs may not adequately address key evidence gaps for payers and HTA bodies nor adequately capture the true value of the PSM technology itself, creating systemic barriers to innovation and adoption. As a result, the clinical implementation of effective PSM solutions is slowed, and patient access is limited.

### The Rationale: Need for a Standardized Value Framework

To overcome key challenges and realize the full potential of PSM, a standardized but adaptable value framework is needed. While specific evidence requirements vary between individual payers and HTA bodies, systematic literature reviews (SLRs) demonstrate that commonalities exist across markets,^19,21,29^ underscoring the feasibility and critical need for consolidated guidance. A unified multi-country value framework elucidating these requirements can begin to align coverage and reimbursement expectations, enabling efficient, high-quality evidence generation despite resource constraints.

### Objective

The primary objective of this research was to construct a novel, conceptual framework that provides an illustrative yet structured and adaptable approach for demonstrating the value of PSM across diverse range of markets and therapeutic areas. To our knowledge, no such comprehensive, stakeholder-informed, and adaptable framework exists in the published literature.

## METHODS

This study used a sequential, mixed-methods approach to identify value drivers, defined as attributes that characterize the value of PSM, through a targeted literature review (TLR), followed by qualitative expert interviews to validate findings and gain stakeholder-specific insights on evaluation criteria and evidence generation requirements for PSM technologies. This integrated approach ensured the framework includes both empirical evidence and practical stakeholder insights, enhancing its robustness and real-world applicability.

### Targeted Literature Review

PubMed search criteria were developed, indexing on the core themes of value demonstration to identify peer-reviewed English-language literature in the PSM space across a range of therapeutic areas (**Supplementary Table S1**); additional hand-searching was performed on the websites of key HTA bodies and major payer organizations (eg, National Institute of Health & Care Excellence, Gemeinsamer Bundesausschuss, US managed care organizations) to identify gray literature and policy documents not indexed in PubMed.

The TLR was conducted using Nested Knowledge^®^, a dedicated literature review software platform. An artificial intelligence (AI) screening model was trained using a comprehensive set of manually screened articles (n = 184) that included primary studies (eg, randomized controlled trials [RCTs], claims analyses, cost-effectiveness analyses), reviews (eg, narrative reviews, SLRs), and gray literature (eg, HTA documents, medical association guidelines, frameworks). The screening model conducted screening of all 9102 titles and abstracts included in the PubMed search and hand-searched articles, enhancing the efficiency of reviewing the large literature base, to provide an advancement probability (a 0-1 probability score predicting the likelihood an article would be included had it been screened manually based off inclusion patterns). The research team manually reviewed articles for relevance sequentially as recommended by the AI screening model, ensuring diverse representation across therapeutic areas, PSM types, and study designs. Additional hand-searching supplemented gaps from the AI model, balancing AI efficiency with rigorous human oversight. Evidence identifying and supporting PSM value themes was extracted from the included literature, with a qualitative assessment of the strength and pertinence of evidence documented for each data point based on relevance, directness, robustness of study type, and demonstration of value drivers. Insights from the TLR were derived within the bounds of predefined value drivers across four themes (clinical, economic, humanistic, and societal), which provided a structure that is consistent with HTA methodologies that recognize the need to assess multiple dimensions of value using diverse study types.

### Qualitative Primary Research

A targeted, stakeholder-stratified sample was recruited to ensure varied representation of clinical decision-makers and reimbursement authorities across key markets, totaling a prespecified quota of 10 participants. This comprised of HCPs (endocrinology/diabetology, infectious diseases, primary care) and 7 payer/HTA representative in the US, UK, and Germany. Participants were recruited through a third-party vendor panel and outreach network to identify eligible participants based on predefined inclusion criteria including familiarity with and historical use of PSM technologies. All participants provided informed consent prior to participation, and institutional review board approval. The vendor followed standard industry practices to ensure compliance with privacy regulations and ethical guidelines. Respondents were selected based on criteria to ensure participants possessed expertise and direct knowledge of current and future PSM technologies within chronic disease management, coverage and reimbursement policies, and evidence generation to demonstrate value for PSM technologies. HCP respondents were included to further capture clinical perspectives of stakeholders directly engaging with patients and the real-world use of PSM technologies. Each participant participated in a 1-hour, double-blind, moderator-led semistructured interview, with all interviews conducted using a web-enabled platform. Interviews focused on understanding market-specific evaluation criteria, perceptions of relevant value drivers derived from the TLR, and evidence generation requirements for PSM technologies. Interview insights were distilled into pooled, consistent themes across stakeholders, and stratified into country-specific findings to inform the framework. Payer/HTAs respondents also provided quantitative weightings of how prespecified value themes currently influence coverage/reimbursement decision-making. The average weighting across payer respondents was calculated to suggest the most important value drivers for framework development.

### Framework Development

Key value drivers identified in the TLR informed the development of a preliminary draft framework. This was tested by HCPs and payers who validated the most important value drivers and provided specific details on the PSM evaluation process in key markets which informed subsequent domains of the framework. Together, these findings informed design of the final conceptual framework which was developed via an iterative process throughout the qualitative primary research phase (**Supplementary Figure S1**).

## RESULTS

### Targeted Literature Review

**Figure 1.**
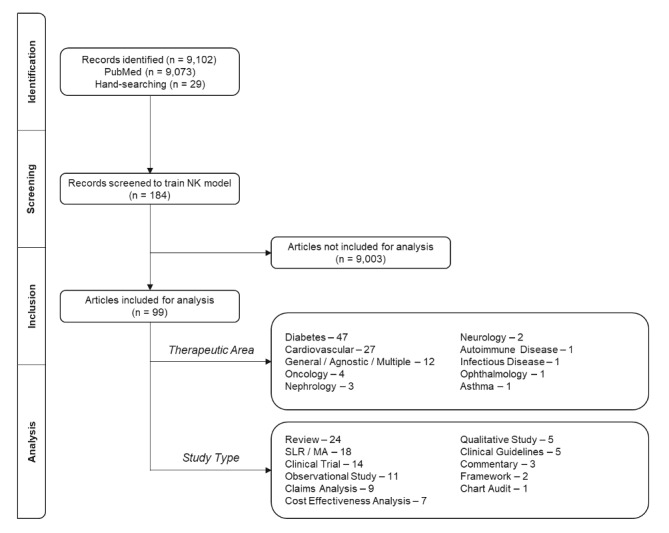
PRISMA Diagram and Breakdown of Therapeutic Areas and Study Types Abbreviations: MA, meta-analysis; NK, Nested Knowledge®; SLR, systematic literature review.

The comprehensive targeted literature search yielded a total of 9102 articles. Of these, 9073 articles were identified through a search strategy conducted in PubMed, and 29 articles were sourced via targeted hand-searching of gray literature, payer guidelines, and HTA documents. A total of 99 articles were included for full data extraction following the AI-enabled screening process. Articles were selected for their high quality and relevance, encompassing a diverse range of therapeutic areas and study designs. The range of study designs included narrative reviews (n = 24), SLRs & meta-analyses (n = 18), clinical trials (n = 14), observational studies (n = 11), claims analyses (n = 9), cost-effectiveness analyses (n = 7), guidelines (n = 5), qualitative studies (n = 5), commentaries (n = 3), frameworks (n = 2), and a chart audit (n = 1) to ensure all aspects of PSM value were captured. The included literature originated from a diversified perspective of payer and HTA evaluation approaches, including the US (n = 38), global/multi-country (n = 33), UK (n = 8), China (n = 4), Spain (n = 2), France (n = 2), Denmark (n = 2), Germany (n = 1), Italy (n = 1), Switzerland (n = 1), Belgium (n = 1), the Netherlands (n = 1), Finland (n = 1), Australia (n = 1), South Korea (n = 1), Taiwan (n = 1), and Thailand (n = 1) to ensure a comprehensive review of payer/HTA criteria, though article inclusion was not limited to these geographies. The evidence landscape remains heavily concentrated in diabetes (n = 47) and cardiovascular (n = 27) therapeutic areas, which account for most published studies and represent the most established applications of PSM, individualized clinical pathways, and reimbursement frameworks. Additional articles explored emerging PSM in therapeutic areas including oncology (n = 4), nephrology (n = 3), neurology (n = 2), asthma (n = 1), infectious disease (n = 1), and ophthalmology (n = 1) (**Figure 1**). **Supplementary Table S2** contains a comprehensive list of included articles.

Evidence requirements and patterns varied across geographies. A plurality of evidence originated from the US. Study design diversity in US evidence included clinical trials (n = 6), claims analyses (n = 5), and cost-effectiveness analyses (n = 2), illustrating payers’ acceptance of a variety of evidence types demonstrating clinical and economic benefit to support coverage decisions. UK evidence patterns strongly emphasized cost-effectiveness analysis alongside clinical outcomes, with clinical trials (n = 3) serving as the primary study design to communicate value.

Value drivers were distributed across the four themes to comprehensively evaluate PSM, comprising clinical value drivers (14 drivers) reflecting outcomes such as treatment efficacy/clinical utility (ie, patient health outcomes with device usage) and diagnostic efficacy (specificity/sensitivity), economic drivers (8 drivers) encompassing outcomes including HCRU, cost-effectiveness, and budget impact, humanistic drivers (7 drivers) evaluating patient-centered care and QoL components, and societal value drivers (12 drivers), recognizing the increasing emphasis on population health, social determinants of health, and the broader societal benefits that healthcare technologies can provide beyond individual patient care (see **Table 1** for definitions of value drivers). This foundation provided a structured approach for assessing PSM technologies that capture a multifaceted value proposition and aligns with current trends in value-based healthcare and health technology assessment.

**Table 1.** Value Driver Definitions

**Value Driver**	**Definition**	**Examples**
Clinical		
Treatment efficacy/ clinical utility	Effectiveness of follow-up treatment(s) with self-monitoring	Reduction of HbA1c with CGM in diabetes; BP control with home BP monitors
Diagnostic efficacy (sensitivity/specificity)	Ability to correctly identify true (or false) positives/negatives	CGM accuracy for hypoglycemia; wearable ECG for arrhythmia
Patient actionability	Extent to which patients can act on test results	Insulin titration based on CGM; antihypertensive adjustment after home BP monitoring
Symptom improvement	Improvement in patient-reported symptoms	Fewer hypoglycemic episodes; reduced asthma attacks with digital inhaler
Behavior change	Change in patient behavior from intervention	Increased glucose checks; lifestyle alterations to reduce risk of event
Treatment adherence	Consistency in following prescribed regimen	CGM wear rates; medication refill rates
Clinical guidelines	Alignment with clinical practice guidelines	ADA/ESC recommendations for CGM/BP monitoring
Monitoring of adverse events	Ability to detect/report/reduce adverse events	Early detection of hypoglycemia; arrhythmia detection via wearable ECG
Treatment choice	Options for personalizing treatment	Clinical choice between CGM and SMBG; digital vs traditional therapy
Early detection*	Identification of disease progression/complications earlier	Home BP for masked hypertension; wearable ECG for atrial fibrillation
Early intervention*	Ability to intervene sooner via monitoring	Prompt therapy after high glucose alert; early atrial fibrillation treatment
Risk of episode/event	Reduction in risk of acute/chronic events	Fewer hospitalizations for heart failure; reduced severe hypoglycemia episodes
Treatment initiation*	Facilitation of starting therapy	Starting insulin after CGM trends for NIIT patients with type II diabetes; antihypertensive after home BP
Safety/risk of self-monitoring	Potential risks or safety concerns associated with self-monitoring devices	Skin irritation from CGM; anxiety from frequent alerts; risk of inaccurate data/QC issues
Economic		
HCRU (healthcare resource utilization)	Impact on healthcare resource use	Fewer ER visits with CGM; reduced outpatient visits
Cost effectiveness	Value for money compared with alternatives	ICER per QALY for CGM vs SMBG; CEA for BP monitors
Budget impact	Financial impact on payer/health system	Annual cost of CGM adoption; impact on medical spend
Quality measure	Performance metrics tracked by payers and health systems to assess care quality and outcomes	HEDIS diabetes quality measures improved with CGM use; achievement of blood pressure control targets with home BP monitoring; reduction in HbA1c levels meeting quality thresholds
Humanistic		
QoL (quality of life)	Effect on patient well-being and functioning	Improved diabetes-related QoL; reduced anxiety with monitoring
Patient convenience	Ease of use and integration into daily life	Fewer finger sticks with CGM; fewer in-person clinic/PCP visits
Treatment frequency^b^	Frequency of associated interventions	Reduced frequency of treatment and/or reduction of overtreatment
Patient comfort	Physical/psychological comfort with intervention	Less pain with sensors; reduced anxiety
Patient satisfaction	Patient satisfaction with monitoring/therapy	High satisfaction associated with “the value of knowing” about ones’ health status
Patient privacy	Protection of patient data and confidentiality	Secure data in digital platforms
Treatment choice	Options available to patients and providers for personalizing treatment selection and delivery	Humanistic choice between therapies based on patient lifestyle (eg, selection of home BP monitoring vs clinic-based monitoring)
Societal		
Health equity	Reduction of disparities in access/outcomes	Improved CGM access in high-risk groups with lower access to healthcare
Data privacy	Safeguarding of personal health information	Encryption, GDPR/HIPAA compliance
Usability	User-friendliness of device/software	High SUS scores; intuitive app interfaces
Capacity constraints	Impact on health system capacity	Reduced burden on healthcare systems with fewer in-person visits
ESG footprint	Environmental, social, governance impact	Device recycling programs; environmental impact of discarding of disposable
Accessibility	Ease of access for all patient groups	Device availability for patients with sub-conditions or comorbidities, or difficulty using devices
Patient-provider relationships	Quality of patient-provider communication	Patient advocacy groups engaging with payers to drive adoption of self-monitoring
Technological innovation	Innovation of self-monitoring devices for indications, biomarkers, or patient groups that previously lacking access to technology	At-home HIV viral load testing
Patient/physician perceptions	Attitudes and beliefs about intervention	Perceived usefulness of CGM; physician confidence that use will improve clinical outcomes
Payer/provider relationships	Collaboration/alignment between payers and providers	Joint guideline development; shared savings programs
Physician oversight	Extent of physician oversight required to effectively manage self-monitoring	Reduction in in-person physician check-ups for HIV viral load testing or postchemotherapy neutrophil counts
Data gaps	Areas where evidence or data is lacking	Lack of long-term outcomes; insufficient pediatric data

Abbreviations: ADA, American Diabetes Association; BP, blood pressure, CEA, cost effectiveness analysis; CGM, continuous glucose monitoring, ECG, electrocardio- gram; ER, emergency room, ESG, environmental, social, and governance; GDPR, General Data Protection Regulation; HbA1c, glycated hemoglobin HEDIS, Health Effectiveness Data and Information Set; HIPAA, Health Insurance Portability and Accountability Act; ICER, incremental cost-effectiveness ratio; NIIT, nonintensive insulin therapy; PCP, primary care physician; QALY, quality-adjusted life years; QC, quality control; QoL, quality of life; SMBG, self-monitoring blood glucose; SUS, System Usability Score.

^a^Concurrently evaluates the economic implications of value drivers (eg, early detection of disease progression reduces long-term hospitalization, reducing healthcare resource utilization).

^b^Concurrently evaluates the economic implications of value drivers (eg, less frequent need of an associated intervention due to patient self-monitoring is likely beneficial for direct pharmaceutical costs).

The analysis revealed considerable variation in evidence strength across the four value driver themes (**Supplementary Figure S2**). Clinical value was the most prominent, with 145 of 299 reported outcomes classified as high-strength, primarily from robust clinical trial data. Prevalent drivers included treatment efficacy/clinical utility, symptom improvement, treatment adherence, monitoring of adverse events, and behavior change. Fifty-nine of 158 economic value drivers were high-strength. Cost-effectiveness analyses and HCRU, captured directly or via secondary endpoints such as hospitalizations and emergency visits, were most commonly reported, though budget impact was less commonly assessed. Humanistic value drivers were less emphasized, with 19 of 78 value drivers classified as high-strength, given they were often reported as secondary endpoints in clinical trials. QoL was the most frequently assessed humanistic outcome, along with patient convenience. Twenty-three of 66 societal value drivers were classified as high-strength. Societal value drivers are often derived from preceding clinical, economic, and humanistic drivers; for example, capacity constraints are likely alleviated due to reduced hospitalizations and HCRU, and improved health equity was inferred from articles most frequently given its increasing emphasis across many payer and HTA bodies.

### Qualitative Primary Research

**Figure 2.**
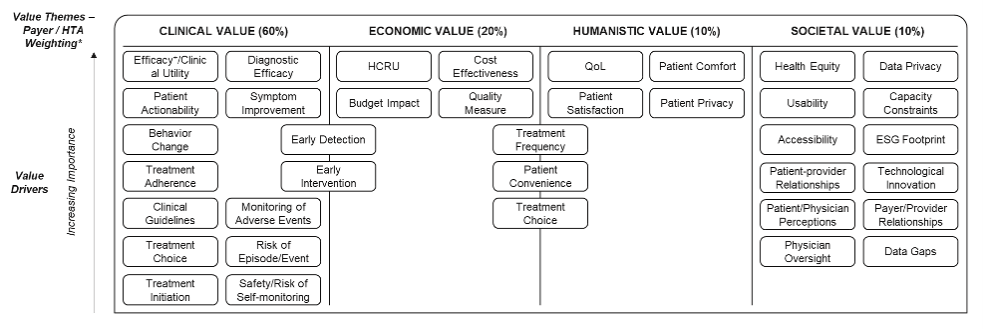
Payer and HTA Weighting of PSM Value Themes and Associated Drivers Abbreviations: ESG, environmental, social, and governance; HCRU, healthcare resource utilization; HTA, health technology assessment; QoL, quality of life; SaMD, software as medical device. *Qualitative primary research respondents (payers only) were asked to quantitatively assign priority weightings to each of the four value themes (N = 3, US; N = 3, UK; N = 1, Germany) +Efficacy assumes patient actionability and refers to patient health outcomes with device usage Value drivers in between sections can be considered from both value themes perspectives (eg, the clinical value of initiating an early intervention to prevent disease progression and the economic value of initiating a cheaper, earlier line of therapy).

Qualitative primary research with 3 HCPs and 7 payer representatives from the US, UK, and Germany validated the findings from the TLR but also revealed that demonstrating value follows distinct market-specific evaluation criteria across healthcare systems. A key finding was the quantified weighting attributed to value drivers in decision-making; on average across participants, payer/HTA respondents allocated 60% of overall emphasis to clinical value, 20% to economic value, and 10% each to humanistic and societal value drivers. The perceived importance of value drivers identified from the TLR by qualitative primary research respondents are represented in **Figure 2**. This weighting provides a suggestion for an initial prioritization for evidence generation activities, ensuring resources are aligned with the most critical decision-making factors for payers. Clinical and economic value drivers were critical, with payers consistently emphasizing the need for evidence on improved patient outcomes and decreased HCRU compared to standard of care. US payers prioritized clinical utility and budget impact within timeframes up to 18 months, UK payers emphasized cost-effectiveness analysis driven by NICE methodology within National Health Service funding constraints, and German payers evaluate coverage decisions based on clinical value assessment which are separate from price negotiations. These findings are encapsulated within the framework to directionally represent the perspectives of payers and HTA across key markets for evidence requirements and solutions that can be further extrapolated to additional markets.

Discussion of evidence generation requirements confirmed that published peer-reviewed data in journals are strongly preferred over unpublished manufacturer-generated studies across all markets. RCTs were considered the gold standard for clinical value demonstration across all geographies, particularly noted by German payers for regulatory assessment. However, both US and UK payers acknowledged and actively utilized pragmatic clinical trials (PCTs) and RWE as important supplementary data to demonstrate real-world impact and support reimbursement decisions. Humanistic and societal value drivers were recognized as important supplementary considerations that can support coverage decisions, enhancing the overall value proposition. Payers acknowledged the value of patient empowerment, QoL improvements, and healthcare system capacity constraint reductions, though these factors remained consistently viewed as secondary to clinical and economic outcomes in reimbursement decision-making processes. The weighting structure provides a systematic approach for evidence generation prioritization, ensuring that most resources are allocated to demonstrating clinical and economic value while maintaining balanced consideration of humanistic and societal impacts that increasingly influence coverage and reimbursement decisions.

### Framework

The synthesis of findings from both the TLR and qualitative primary research culminated in the development of a comprehensive framework structured around three interconnected pillars: Value Drivers, Evaluation Criteria, and Evidence Generation Considerations, which collectively provide a structured approach for developing the evidence for and demonstrating the value of PSM across diverse markets and therapeutic areas. The value drivers represent the most critical considerations comprising clinical, economic, humanistic, and societal value across all evaluation contexts to ensure the value of PSM can be appropriately assessed by payers and HTA bodies (**Figure 2**). Evaluation criteria represent market-specific assessment approaches that guide payer and HTA decision-making processes derived from the qualitative primary research. The evaluation criteria categories derived from the key market considerations are (1) clinical assessment, (2) cost-effectiveness, (3) budget impact, and (4) comparative pricing, each incorporating distinct elements of the evaluation criteria by payers and HTA bodies as shown in **Supplementary Figure S3**. Evidence generation considerations encompass the methodological approaches required to demonstrate value, including RCTs, PCTs, RWE, economic modeling approaches, and other health economics and outcomes research study types as shown in **Supplementary Figure S4.** These evidence types are strategically aligned with specific value drivers and evaluation criteria to ensure that the most appropriate and robust evidence is generated to support coverage and reimbursement decisions. Recommended evidence generation activities have been validated by qualitative primary research respondents for specific market use cases and may include a combination of study types to best address payer/HTA requirements.

**Figure 3.**
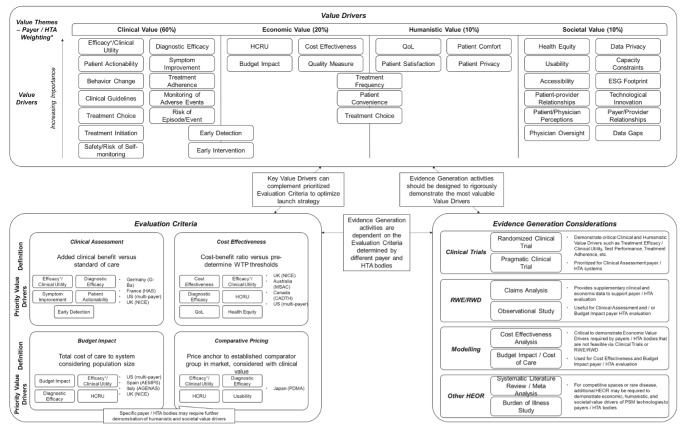
A Conceptual Framework for PSM Value Demonstration +Efficacy assumes patient actionability and refers to patient health outcomes with device usage (ie, treatment efficacy). Abbreviations: AEMPS, Agencia Española de Medicamentos y Productos Sanitarios; AGENAS, Agenzia Nazionale per i Servizi Sanitari Regionali; CADTH, Canadian Agency for Drugs and Technology in Health; ESG, Environmental, Social, and Governance; G-BA, Gemeinsamer Bundesausschuss (Federal Joint Committee); HAS, Haute Autorité de Santé (National Authority for Health); HCRU, healthcare resource utilization; HEOR, health economics and outcomes research; HTA, health technology assessment; MSAC, Medicare Services Advisory Committee; NICE, National Institute of Health & Care Excellence; PDMA, Pharmaceuticals and Medical Devices Agency (Dokuritsu Gyōseihōjin Iyakuhin Iryōki-ki Sōgō Kiki); QoL, quality of life; RWD, real-world data; RWE, real-world evidence.

The framework operates as an interconnected system where key value drivers are accentuated for individual payer/HTA evaluation criteria, which can then be demonstrated through relevant evidence generation considerations, creating a dynamic relationship that allows for market-specific evidence generation strategies while maintaining consistency in value demonstration approaches, as shown in **Figure 3**. This interconnectedness ensures that the framework can accommodate the varying requirements of different healthcare systems while providing a directional approach to identifying the most relevant value drivers and corresponding evidence needs for PSM technologies. The framework’s flexibility enables its application across both established and emerging indications, while maintaining relevance for diverse PSM modalities ranging from CGM to home blood pressure monitoring to viral load testing devices.

## DISCUSSION

The framework was developed using a mixed-methods approach, which integrated systematic insights from a TLR with nuanced, real-world perspectives from key stakeholders. The TLR identified a wide array of value drivers, ensuring foundational breadth, while the qualitative interviews prioritized these drivers to reveal market-specific evaluation criteria and evidence generation requirements, thus anchoring the framework in practical stakeholder needs. This dual methodology roots the framework in both academic literature and practical stakeholder expertise.

### Using the Framework

The framework is intended to be used by a variety of stakeholders to provide guidance on how the value of PSM in chronic diseases can be demonstrated effectively to payers and HTA bodies across several markets. This framework proposes best practices for evidence generation and value demonstration, offering flexibility across a variety of emerging PSM technologies and varying market requirements. By synthesizing these diverse perspectives, it offers a hypothesis for advancing PSM technology development and securing patient access.

Concurrently, the framework will guide developers in planning and generating targeted evidence that is aligned with stakeholder expectations, optimizing resource allocation and maximizing the utility of evidence. Crucially, the framework will support payers and HTA bodies in establishing transparent and appropriate coverage criteria based on consistent, evidence-based evaluation methodologies, thereby fostering broader adoption and improving patient access.

**Figure 4.**
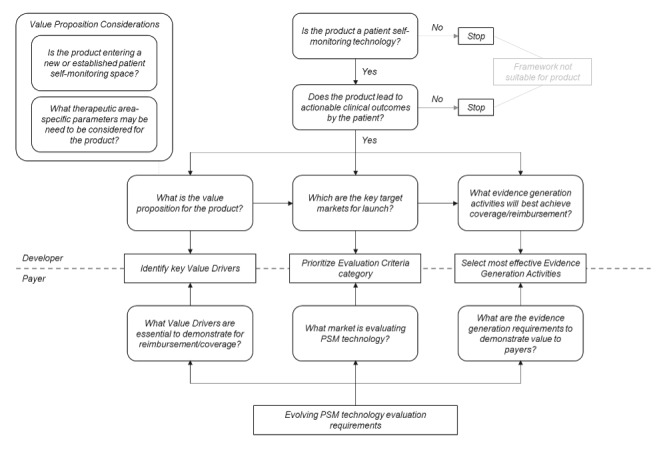
A Conceptual Workflow for Using the PSM Value Demonstration Framework Abbreviation: PSM, patient self-monitoring.

The framework is not intended to replace integrated evidence generation plans by manufacturers. To ensure the framework components can be accurately navigated, it is essential that an initial value proposition exists and foundational evidence generation activities have been completed to robustly demonstrate clinical validity (ie, sensitivity and specificity) in representative patient groups (**Figure 4)**. In addition, payers can use the framework to consider what value drivers and evidence generation activities are most important to satisfy reimbursement and coverage requirements.

### Positioning of Framework in Literature

Increased regulatory and payer attention to PSM technologies creates market pressure for more robust guidance and expectations,^30,31^ which in turn drives framework adoption as stakeholders seek standardized evidence approaches for product evaluations. Commercial viability depends on meeting evolving stakeholder expectations, as payers increasingly tie coverage and reimbursement to demonstrated real-world value and improvement of patient outcomes in addition to traditional clinical endpoints alone.^32,33^ Risk mitigation for both developers and payers is facilitated through framework adoption, as standardized evidence requirements reduce uncertainty about what constitutes adequate evidence for PSM technologies.

The framework has been developed to accommodate a variety of methods for evidence generation and therefore can be utilized within emerging therapeutic areas and use cases such as oncology, asthma, rheumatology, and chronic inflammatory bowel conditions (ie, any chronic condition in which manifestations of acute events predictable by biomarkers can be mitigated by patient actionability). The goal of the framework is to provide an initial structured, adaptable, and stakeholder-validated methodology to articulate and synergize the value proposition of emerging PSM technologies.

### Practical Use of Framework

**Figure 5.**
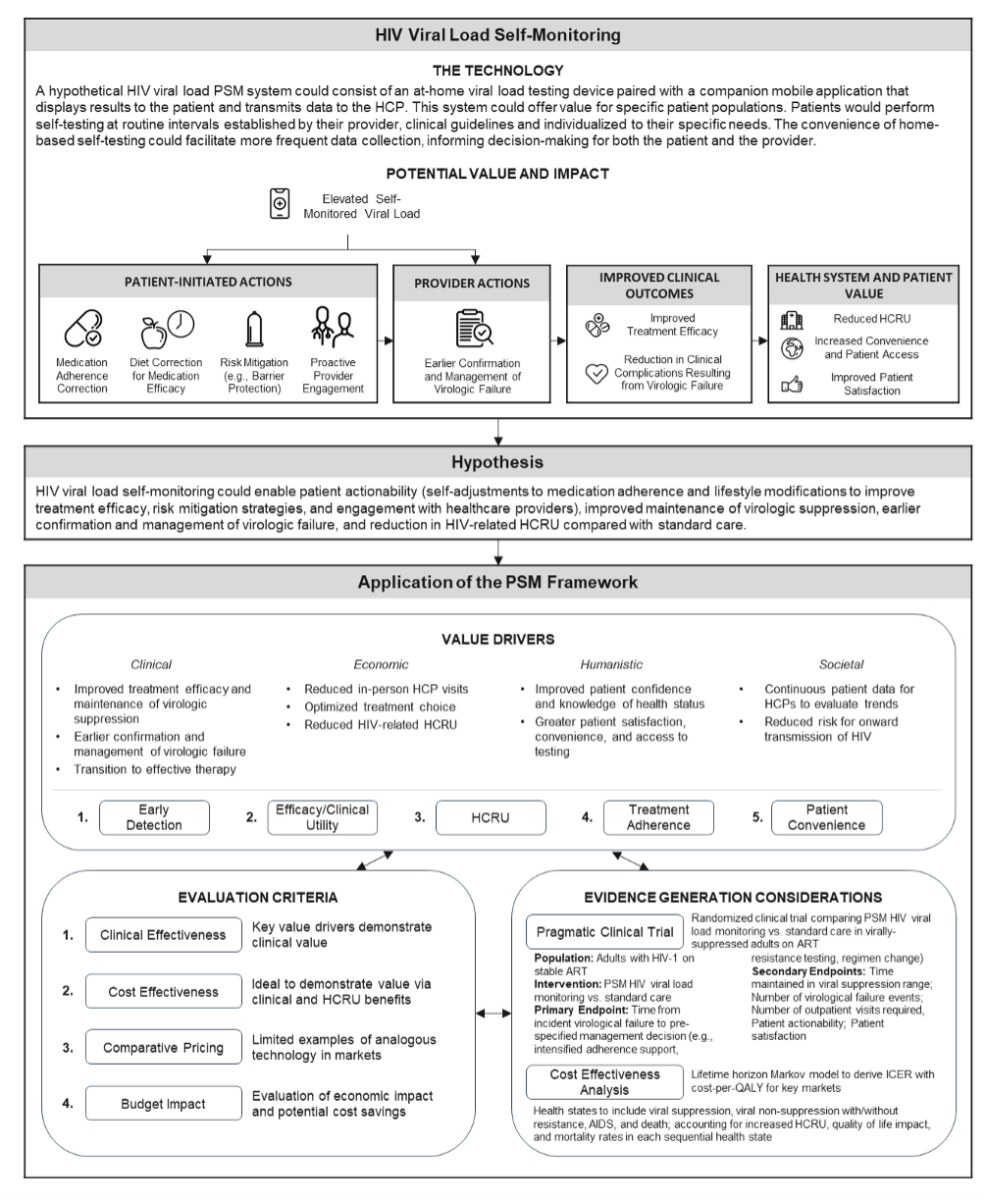
Hypothetical Example Application of the Value Framework: Case Study of HIV Viral Load Self-Monitoring Abbreviations: ART, antiretroviral therapy; HCP, healthcare practitioner; HCRU, healthcare resource utilization; ICER, incremental cost-effectiveness ratio; PSM, patient self-monitoring; QALY, quality-adjusted life-year.

The framework is designed to guide multiple stakeholders, including developers, payers, HTA bodies, and HCPs, in understanding how PSM demonstrates value across different contexts. Organizations planning comprehensive evaluation of PSM technologies should prioritize strategic evidence generation that acknowledges dynamic, differentiated approaches rather than prescriptive methodologies. A conceptual case study has been represented in **Figure 5**, showcasing a hypothetical futuristic example of PSM of HIV viral load monitoring. Development of a PSM technology that allows patients with HIV to self-monitor viral load could lead to earlier detection of increased viral load, sustained viral suppression without resistance, and reduced HCRU. Considering these characteristics, the most optimal evaluation criteria that considers the key value drivers of early detection, treatment efficacy/clinical utility, and (reduced) HCRU are clinical assessment and cost-effectiveness. Simulation through the framework indicates a PCT and cost-effectiveness model would best capture the value of this PSM technology, with suggested endpoints maximizing the effectiveness of value communication to payers/HTA bodies. The interconnectivity between the value drivers and the prioritized evaluation criteria leads to effective evidence generation recommendations, underpinning how this framework captures value of PSM for coverage and reimbursement decision-making.

### Wider Implications for PSM Utilization

While the framework primarily serves as an initial basis to consider the value of PSM from a coverage and reimbursement perspective, wider stakeholder engagement and further resting is critical to ensure the continued innovation and real-world adoption of PSM technologies. HCPs play a central role in guiding patient-clinician interactions, ensuring that patient-led healthcare decisions translate into optimal clinical outcomes. It is therefore essential for HCPs to be actively involved in patients’ PSM use to ensure clinical outcomes are sustained long-term (ie, avoid patient data fatigue or overinterpretation of results) and have the functionality to re-establish primary care should a patient fail to appropriately action their healthcare needs, though a robust interface to manage HCP demand and capacity is required.^34^

### Limitations

The current emphasis on chronic diseases in this framework may reflect the market-driven availability of established devices and the supporting literature rather than be an optimized framework design for capturing full reimbursement potential across therapeutic areas. This therapeutic area bias toward diabetes and cardiovascular diseases may limit framework applicability to other domains including acute care and preventive medicine, potentially creating evidence gaps that do not demonstrate broader economic and clinical impact. However, this equally highlights the limitations within current payer and HTA evaluation criteria and metrics, in which clinical or economic value is captured via conventional methodologies that underestimate key drivers of the value of PSM (eg, the value of knowing, patient empowerment, improved self-management, and behavioral changes), and this framework is a first step toward highlighting this. The small qualitative sample size further limits this study, as the primary research was restricted to interviews with a limited number of stakeholders across only three key markets (US, UK, Germany). As a result, the findings may not fully capture the diversity of perspectives or market-specific nuances present in other regions, particularly from low- and middle-income countries, and markets with different reimbursement mechanisms. Quantitative conclusions derived from the research and insights from the framework should therefore be considered directional rather than wholly generalizable to a global audience. Future research should aim to validate this framework’s applicability in additional settings, test its utility for specific emerging PSM technologies, and conduct a larger quantitative survey to confirm the payer value driver weightings across a broader range of markets. Furthermore, patient voice was a key element of PSM value that the study methodology was unable to capture. Elements of this are considered via societal value drivers such as usability and accessibility; however, it is critical to further explore the patient perspective to support this and integrate into the framework in future work to expand its robustness and comprehensiveness. Additionally, while the framework provides a structured approach for evaluating PSM, it is not intended to replace the need for individualized integrated evidence generation plans. Each product will still require tailored planning to address the unique clinical, regulatory, and market access requirements relevant to its specific launch strategy and target indications.

## CONCLUSIONS

PSM represents a broadening healthcare modality that requires thoughtful evidence generation to adequately demonstrate their value to a diverse set of stakeholder groups and healthcare systems. Unlike traditional medical interventions, these technologies operate at the intersection of clinical care and patient empowerment, necessitating evidence frameworks that capture both treatment efficacy/clinical utility and real-world implementation outcomes.

Prior to this work, no evaluation framework existed to thoroughly articulate the value of PSM. This study used mixed methodology to comprehensively evaluate existing evidence generation activities in the PSM space, incorporating perspectives from both payers and HCPs to provide a holistic understanding of stakeholder requirements and implementation challenges. This study found substantial variability in evidence quality and gaps in patient-centered outcome measurement, highlighting the need for more systematic approaches to evidence generation.

This framework provides an initial structured foundation for stakeholders to consider various evidence generation activities and requirements across key markets, enabling more strategic evidence generation planning and improved articulation of value in innovative PSM technologies. The framework acts as a first step to address the critical need for consistent evidence standards while illustrating dynamic, differentiated approaches to evidence generation that will streamline innovation and evaluation strategies. By enabling PSM to enhance patient autonomy and support HCPs in delivering better access to care, the framework can provide holistic value across the healthcare ecosystem.

### Disclosures

The authors declare no potential conflicts of interest.

## Supplementary Material

Online Supplementary Material
