# Supplementary material for: Unlocking the Value of Patient Self-Monitoring Technologies: A Multi-Country Framework to Calibrate Payer and HTA Coverage and Reimbursement Considerations with Innovation
Source: J Health Econ Outcomes Res. 2026 Jun 16;13(1):248–54. doi: 10.36469/001c.163078 (PMC13278614; doi:10.36469/001c.163078)
Supplement: Online Supplementary Material [file jheor_2026_13_1_163078_349880.pdf]

Journal of Health Economics and Outcomes Research

Online Supplementary Material

Unlocking the Value of Patient Self-Monitoring Technologies: A Multi-Country Framework to Calibrate Payer and HTA Coverage and Reimbursement Considerations with Innovation. *JHEOR*. 2026;13(1):248-254. doi:10.36469/jheor.2026.163078

PSM Evidence Requirement Disparity 1

Targeted Literature Review Methodology 5

Qualitative Primary Research Methodology 6

Hypothetical Example Application of the PSM Value Demonstration Framework: A Case Study of HIV Viral Load Self-Monitoring 7

REFERENCES 9

This supplementary material has been provided by the authors to give readers additional information about their work.

This is an open-access article distributed under the terms of the Creative Commons Attribution 4.0 International License (CCBY-4.0). View this license's legal deed at http://creativecommons.org/licenses/by/4.0 and legal code at http://creativecommons.org/licenses/by/4.0/legalcode for more information.

PSM Evidence Requirement Disparity

PSM technology is an emerging modality that allows patients to take a more active role in their health, which in turn helps alleviate dependency on HCPS – easing the strain on health systems facing a rising burden of chronic disease.[1] However, despite the success of established PSM technologies such as CGM for diabetes and INR monitoring for cardiovascular diseases, there lacks a standardized framework for PSM technologies to demonstrate value and secure reimbursement in other therapeutic areas.[2-5] The authors identified key markets to comprehensively represent global reimbursement/coverage methodologies, consisting of the US, UK, Germany, Italy, Australia, and Japan, given their varied methods of assessing value and redundancies with other markets (e.g., Canada, France, Spain, etc.).

In the US, following FDA clearance or approval (e.g., via the 510(k) pathway), developers negotiate coverage and payment with individual payers. The Centers for Medicare & Medicaid Services (CMS) establishes Medicare payment rates that often serve as a benchmark for commercial price negotiations (e.g., contracts indexed to a percentage of the Medicare rate), with commercial payers generally making coverage decisions using clinical and economic evidence relevant to their member population and benefit designs. For many emerging technologies, CMS does not issue a National Coverage Determination (NCD). In these cases, coverage is set through Local Coverage Determinations (LCDs) developed independently by each Medicare Administrative Contractor (MAC), which can result in regional variation where some MACs cover a technology while others do not, creating a fragmented coverage landscape for developers to traverse. This is particularly pertinent to innovation in emerging therapeutic areas and modalities that is yet to demonstrate high-impact value for patients. The US market represents a heterogenous coverage environment consisting of federal public programs for distinct

populations (i.e., Medicare for adults >65 years old and certain patient populations, and Medicaid for many low-income individuals) operating alongside a large, diverse private payer sector. Coverage policies therefore vary across individual payers, each of which considers clinical and economic value with different perspectives.[6]

To gain UK reimbursement, developers must obtain Medicines and Healthcare products Regulatory Agency (MHRA) approval or UK Conformity Assessed (UKCA) mark to demonstrate compliance with medical device regulations (a CE mark continues to be accepted in the UK until 2028/2030 depending on device type). Under NICE's newly consolidated HealthTech Evaluation program – combining the previous Medical Technologies Evaluation, Interventional Procedures, and Diagnostic Assessment routes – PSM technologies are assessed based on cost-utility (i.e., considers costs and QALYs) and cost-comparison (i.e., considers costs only) analyses based off a pre-submitted evidence dossiers. Although NICE provides a centralized reimbursement recommendation, funding, commissioning, and adoption of PSM technologies are largely devolved to integrated care boards (ICBs) and/or NHS England, leading to variation in access between areas, reinforcing the need for a compelling value proposition for PSM technologies.[7]

In Germany, a CE mark must be obtained to demonstrate conformity with EU regulations. Reimbursement within the statutory health insurance system then proceeds through centralized listing in either the Bundesinstitut für Arzneimittel und Medizinprodukte (BfArM) Digitale Gesundheitsanwendungen (DiGA) directory for digital health applications or the Hilfsmittelverzeichnis for hardware-based devices, with the Spitzenverband Bund der Krankenkassen (GKV-Spitzenverband) evaluating clinical outcomes, patient-relevant benefits, and safety. For DiGA, the developer negotiates the reimbursement price directly with the GKV-

Spitzenverband following provisional listing, and for Hilfsmittelverzeichnis, pricing is determined through fixed amounts or negotiated contracts established by the GKV-Spitzenverband. Unlike systems driven by strict cost-effectiveness threshold, the German pathway emphasizes evidence of clinical benefit and structural healthcare improvements to establish reimbursement eligibility and inform price negotiations.[8]

In Italy, reimbursement for medical devices has historically been fragmented, however the recent Programma Nazionale di Health Technology Assessment (PNHTA) now empowers the Agenzia Nazionale per i Servizi Sanitari Regionali (AGENAS) to provide evaluations and recommendations on the use of medical devices, including PSM technologies. Livelli Essenziali di Assistenza (LEA) are national decrees that define coverage and accessibility, based off clinical effectiveness and budget impact. Developers then engage the regional and local healthcare agencies, the Azienda Sanitaria Locale (ASL) or Agenzia di Tutela della Salute (ATS), with prices determined via public tender, which are increasingly aggregated at a regional level via public buyers within the regional and local health authorities of the Servizio Sanitario Nazionale (SSN).[9]

Australia employs a rigorous, evidence-based HTA approach for reimbursement of PSM technologies. Following market authorization by the Therapeutic Goods Administration (TGA), developers must submit a dossier to the appropriate HTA body based on the technology's funding stream. For medical services and many non-implantable technologies seeking funding under the Medicare Benefits Schedule (MBS), the Medical Services Advisory Committee (MSAC) conducts the assessment. This process typically involves a PICO confirmation by the PICO Advisory Sub-Committee (PASC) and a detailed critique of clinical and economic evidence by the Evaluation Sub-Committee (ESC) before MSAC provides a recommendation to

the Minister for Health. Alternatively, products intended for direct subsidy – most notably Continuous Glucose Monitoring (CGM) devices – are funded through the National Diabetes Services Scheme (NDSS). While historically linked to the Pharmaceutical Benefits Advisory Committee (PBAC), assessment responsibility for diabetes technologies has increasingly shifted to MSAC to ensure a consistent evaluation of comparative safety, clinical effectiveness, cost-effectiveness, and total budget impact. Successful listing requires demonstrating value against current standard of care, often necessitating price negotiations to meet implicit cost-effectiveness thresholds (typically between AUD 45,000 – AUD 75,000) before a federal subsidy is granted.[10]

In Japan, medical devices must first obtain regulatory approval (shonin) from the Ministry of Health, Labour and Welfare (MHLW), based on a scientific review conducted by the Dokuritsu Gyōseihōjin Iyakuhin Iryōki-ki Sōgō Kiki (Pharmaceuticals and Medical Devices Agency; PMDA). To ensure marketability, the device's indication should align with a specific Iryō Kiki Meimei Taisei (Japanese Medical Device Nomenclature; JMDN) code. Following regulatory approval, developers apply for reimbursement coverage under the Kokumin Kenkō Hoken (National Health Insurance; NHI). This application is assessed by the Chuikyo (Central Social Insurance Medical Council) within the MHLW to determine the appropriate pricing category: A1/A2 (inclusive/bundled within existing technical fees), B (specific product reimbursement for materials), or C1/C2 (new functional categories for innovative devices, where C2 may establish a new technical fee). Pricing for new categories often references benchmarks from the US, UK, France, Germany, and Australia, utilizing formulas to adjust for price differentials and applying premiums for innovation or clinical utility. Upon Chuikyo approval, reimbursement prices are listed on the NHI tariff, with updates typically published quarterly. Japan provides broad

reimbursement for PSM technologies like CGM, standing alongside other APAC markets such as Australia and South Korea in establishing funded access for these devices.[11]

Overall, as expected, there is a wide degree of heterogeneity in the approach to PSM technology reimbursement assessment. However, most HTA and payers do not have an established framework to effectively demonstrate value for PSM technologies. Some markets have specific guidance for the reimbursement of CGM in diabetes, which underlies the necessity for a differentiated approach to value demonstration for PSM technologies that may not be adequately represented by traditional methods. This underscores the need for a value framework to align developers' evidence generation strategies and payer/HTA approach to evaluating such technologies.

Targeted Literature Review Methodology

As described in the methods section, a TLR was performed to consider the current literature base of the evaluation of PSM technologies. PubMed was used as the sole database of the search given this review was intended to gain a holistic understanding of the existing peer-reviewed literature rather than a systematic, global assessment of all evidence. To this end, a wide search string was constructed to identify articles that represent a diverse range of facets of value for PSM technologies, including a variety of article types including RCT reports, RWE, SLRs and MAs, observational studies, narrative reviews, and cost effectiveness analyses, etc. (**Table S1**). To ensure value could be captured via a payer lens to aid construction of the framework, additional materials were sourced via HTA and payer organization websites to supplement the peer-reviewed literature base. This included grey literature such as HTA documents, medical association guidelines, and frameworks. Articles were not included if they were duplicative of included articles (e.g., two similar RCTs assessing CGM devices with the same endpoints), did

not adhere to the authors' definitions of PSM (e.g., digital health technologies), or did not adequately demonstrate the value of a PSM (e.g., did not establish a link between PSM and clinical outcomes or economic value, etc.).

The Nested Knowledge® platform was used to enhance the efficiency of title/abstract review of 9102 unique articles. The AI screening model was strained using a set of 184 manually screened articles which then generated an "advancement probability" to identify the most relevant articles in the remaining literature, of which the full text were manually screened for inclusion in descending order of advancement probability. Additional hand-searching was used to supplement gaps identified by the authors in the holistic value demonstration of PSM technologies resulting in the final 99 articles included in the analysis.

Included articles were extracted into an evidence grid created in Microsoft Excel. Publication characteristics and study details were extracted, including article type, country/location, and therapeutic area. Value Drivers, as shown in **Figure 2**, were extracted from articles, both those demonstrated directly or implicitly, along with the specific data points or supporting evidence and a qualitative assessment of strength of the claim. A total of 622 value drivers were extracted that informed the construction of the qualitative primary research discussion guide and early iterations of the framework.

Qualitative Primary Research Methodology

Qualitative primary research was used to validate findings from the TLR and further elucidate the perspectives of payers and HCPs across key markets to corroborate and legitimize the framework. Respondents were screened using an external, unbiased vendor, to ensure respondents had sufficient experience working at relevant payer/HTA bodies (e.g., NICE, G-BA,

US MCOs, etc.) or practicing relevant medical specialties (diabetology, endocrinology, infectious disease, internal medicine/primary care/general practice) along with experience and knowledge of current and future PSM technologies. To represent the aforementioned key markets, respondents were sampled to fill a quota of a total of 7 payers from the US, UK, and Germany, and a total of 3 HCPs from the US, UK, Germany, with a primary medical specialty of diabetology/endocrinology, infectious disease, and internal medicine/primary care/general practice. A professional moderator conducted interviews using a pre-prepared discussion guide. Given the heterogeneity in respondents, interviews were semi-structured to give freedom to the respondents' specific insights while ensuring consistency in validation of the TLR findings and testing of initial framework components. Given the research did not index on specific medicinal or therapeutic products or medical devices, there was zero risk to human research subjects thus no IRB exemption status was sought.

Payer respondents were asked to provide quantitative weightings to value themes prioritizing what influenced coverage and reimbursement decisions. An average across payer respondents was calculated and rounded to the nearest 5%.

Figure S2 highlights the evidence generation considerations component of the framework that is incorporated into **Figure 3** in the main text.

Hypothetical Example Application of the PSM Value Demonstration Framework: A Case Study of HIV Viral Load Self-Monitoring

To demonstrate the real-world usability of the framework in context, a hypothetical case study was constructed to illustrate the value of a futuristic PSM technology for HIV viral load monitoring. The current management of HIV involves the use of antiretroviral therapy (ART) to

maintain viral suppression with patients typically attending in-person laboratory viral load testing every 6-12 months, or as recommended by the treating provider (for example, newly diagnosed patients may require viral load testing every 3-4 months). Transient viremia is defined as a temporary increase in patients' viral load. Virologic failure is defined as a persistent, confirmed viral load of >200 copies/mL which may necessitate physician-directed management such as adherence training, increased viral load monitoring, or regimen change upon detection of resistance via genotype testing. The hypothetical PSM technology presented in Figure 5 involves patients performing at-home viral load testing at routine intervals established by their provider and individualized to their specific needs. The primary value proposition of this PSM over standard care is to enable patient actionability (self-adjustments to medication adherence and lifestyle modifications to improve treatment efficacy, risk mitigation strategies, and engagement with healthcare providers), improved maintenance of virologic suppression, and earlier confirmation and management of virologic failure, which may lead to reduction in HIV-related HCRU compared with standard care. To this end, the key value drivers identified for the PSM are 1. early detection, 2. efficacy/clinical utility, 3. HCRU, 4. treatment adherence, and 5. patient convenience. The evaluation criteria this aligns with most directly is cost effectiveness, requiring evidence that early detection translates into meaningful reductions in downstream costs – including avoided complications, hospitalizations, and complex regimen changes – that offset the cost of frequent home testing. To substantiate this, developers could prioritize the following evidence generation activities: (1) a pragmatic randomized trial comparing the hypothetical PSM versus standard care and (2) a cost-effectiveness analysis using decision-analytic modelling (e.g., a Markov model) to quantify the ICER as a cost per QALY gained, incorporating inputs on test performance, probabilities of virologic failure, costs of failure-related events, and patient utility.

A supplementary budget-impact model could demonstrate the net system-wide cost implications over 1-3 years, highlighting savings from avoided clinic visits and complications relative to device costs, thereby addressing payer concerns about short-term affordability. This case study illustrates the practical utility of the framework in translating a PSM technology's' value proposition into a structured evidence strategy aligned with target payer priorities. In this example, the framework clarified that Cost Effectiveness, rather than clinical efficacy alone, is the primary lever for demonstrating value – a distinction that may otherwise be obscured without explicit consideration of payer decision-making processes across geographies. Moreover, the framework provides a consistent pathway for prioritizing evidence generation. Developers can focus resources on the specific evidence types (PCTs, cost-effectiveness modeling, budget-impact analyses, etc.) that directly address the identified evaluation criteria. This targeted approach streamlines efforts, accelerates time to market, and increases the likelihood of securing reimbursement across multiple health systems. For PSM technologies, where clinical and economic value propositions are often novel and heterogeneous across regions, this structured alignment between value drivers, evaluation criteria, and evidence generation represents a critical tool for translating innovation into sustainable market access and patient benefit.

REFERENCES

1. Watson KB, Wiltz JL, Nhim K, Kaufmann RB, Thomas CW, Greenlund KJ. Trends in multiple chronic conditions among US adults, by life stage, Behavioral Risk Factor Surveillance System, 2013-2023. *Prev Chronic Dis*. 2025;22:E15. doi:10.5888/pcd22.240539

2. Aleppo G, Hirsch IB, Parkin CG, et al. Coverage for continuous glucose monitoring for individuals with type 2 diabetes treated with nonintensive therapies: an evidence-based approach to policymaking. *Diabetes Technol Ther*. 2023;25(10):741-751. doi:10.1089/dia.2023.0268

3. 7. Diabetes technology: standards of care in diabetes-2025. *Diabetes Care*. 2025;48(1 Suppl 1):S146-s166. doi:10.2337/dc25-S007

4. Shimbo D, Artinian NT, Basile JN, et al. Self-Measured Blood Pressure Monitoring at Home: A Joint Policy Statement From the American Heart Association and American Medical Association. *Circulation*. 2020;142(4):e42-e63. doi:10.1161/cir.0000000000000803

5. Albrecht R. MCOs following innovative CMS remote monitoring initiative: self-testing service is designed to lower costs and improve outcomes for anticoagulation therapy. *Manag Care Q*. Winter 2006;14(1):13–6.

6. Team NSIS. Reimbursement Knowledge Guide for Medical Devices. Accessed September 4, 2025. https://seed.nih.gov/sites/default/files/2024-01/Reimbursement-Knowledge-Guide-for-Medical-Devices.pdf

7. NICE. NICE technology appraisal and highly specialised technologies guidance: the manual. Accessed December 1, 2025. https://www.nice.org.uk/process/pmg36/resources/nice-healthtechnology-evaluations-the-manual-pdf-72286779244741

8. Ivandic V. Requirements for benefit assessment in Germany and England - overview and comparison. *Health Econ Rev*. 2014;4(1):12. doi:10.1186/s13561-014-0012-8

9. Conti FM. The EU HTA Regulation and the Italian HTA for Medical Devices Plan 2023-2025. ISPOR Europe 2023. Accessed September 4, 2025. https://www.ispor.org/docs/default-source/euro2023/14112023francescocontipresentationispor.pdf?sfvrsn=855105f0_0

10. MSAC. Accessed September 4, 2025. https://www.msac.gov.au/

11. Gross A. Japan Medical Device Reimbursement Update 2024. MedTech Intelligence. Accessed September 4, 2025. https://medtechintelligence.com/feature_article/japan-medical-device-reimbursement-update-2024/

Figure S4. Framework Development Process

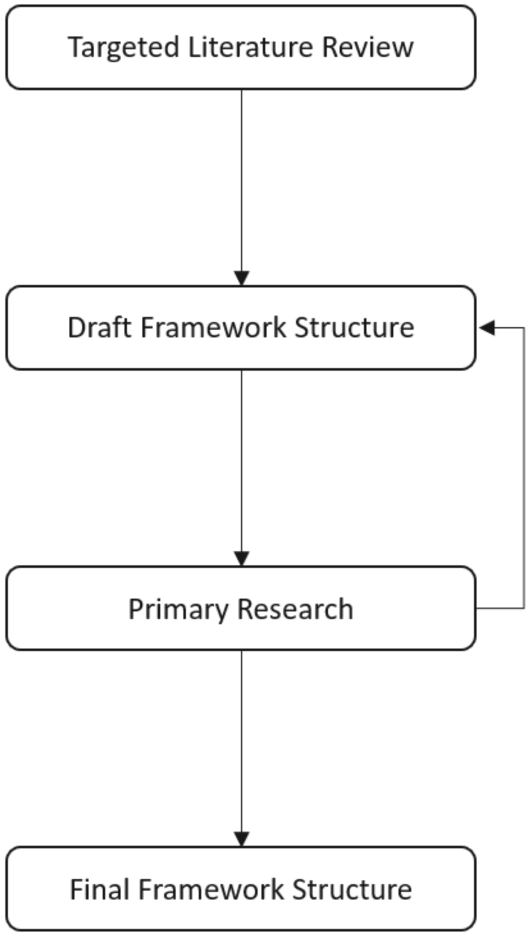

Figure S2. Qualitative Evaluation of Strength of Evidence of Value Drivers Identified in the TLR

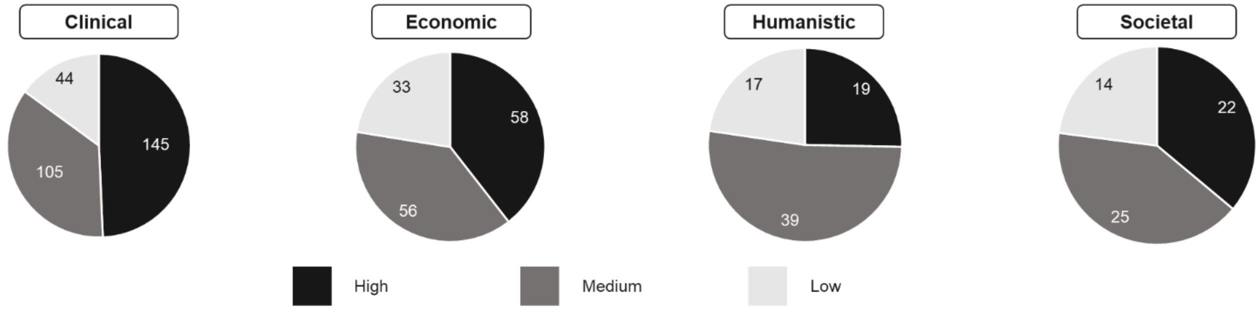

Figure S3. Key Evaluation Criteria and Prioritized Value Drivers for Payer / HTA Reimbursement

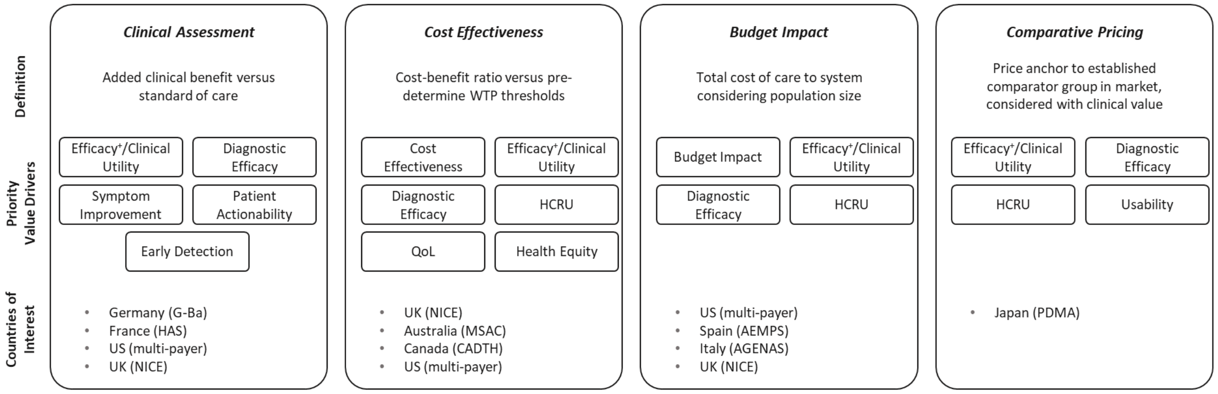

Abbreviations: AEMPS: Agencia Española de Medicamentos y Productos Sanitarios, AGENAS: Agenzia Nazionale per i Servizi Sanitari Regionali, CADTH: Canadian Agency for Drugs and Technology in Health, G-BA: Gemeinsamer Bundesausschuss (Federal Joint Committee), HAS: Haute Autorité de Santé (National Authority for Health), ICER: Incremental Cost-effectiveness Ratio, MSAC: Medicare Services Advisory Committee, NICE: National Institute of Health & Care Excellence, PDMA: Pharmaceuticals and Medical Devices Agency (Dokuritsu Gyōseihōjin Iyakuhin Iryōki-ki Sōgō Kiki), QALY: Quality-adjusted Life Year, VBC: Value-based Care, WTP: Willingness to Pay

Fig. S4 Evidence Generation Activities to Demonstrate the Value of PSM Technologies

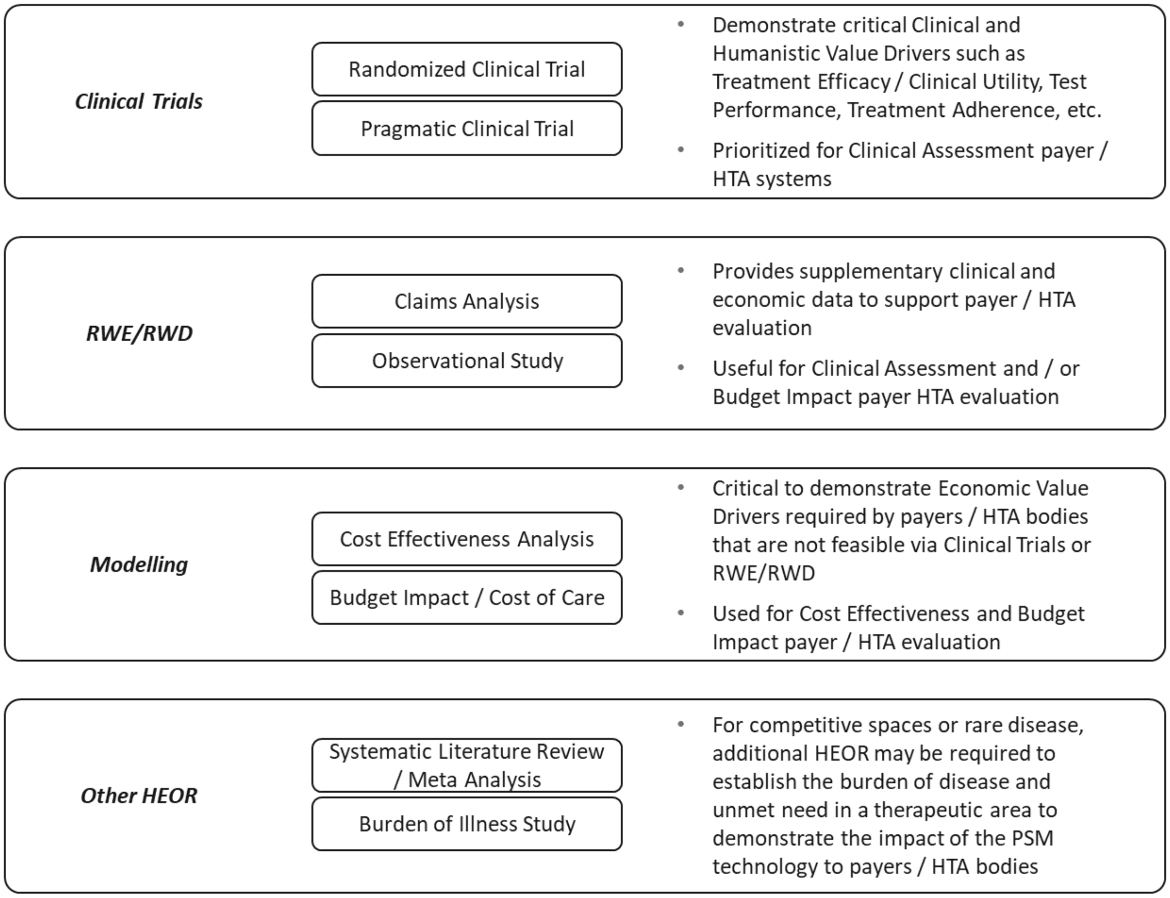

Abbreviations: HEOR: Health Economics and Outcomes Research, HTA: Healthcare Technology Assessment, PSM: Patient Self-Monitoring, RWD: Real-world Data, RWE: Real-world Evidence

Table S1. PubMed Search String

No.	Category	Query	Results (4/3/2025)
1	**Self-monitoring**	"self-examination"[MeSH] OR "self-management"[MeSH] OR "self-monitor"[TiAb] OR "self-monitor"[TiAb] OR "home monitor"[TiAb] OR "self-tracking"[TiAb] OR "self-tracking"[TiAb] OR "continuous monitor"[TiAb] OR "self-examination"[TiAb] OR "self-examination"[TiAb] OR "self-management"[TiAb] OR "self-management"[TiAb] OR "portable device"[TiAb] OR "portable monitor"[TiAb] OR "self-testing"[MeSH] OR "self-testing"[TiAb]	53,337
2	**Clinical utility/ Evidence needs**	"clinical utility"[TiAb] OR "clinical validity"[TiAb] OR "analytical validity"[TiAb] OR "analytical performance"[TiAb] OR "scientific validity"[TiAb] OR "clinical performance"[TiAb] OR "evidence generation"[TiAb] OR "evidence strategy"[TiAb] OR "evidence needs"[TiAb] OR "access strategy"[TiAb] OR "coverage"[TiAb] OR "adoption"[TiAb] OR "reimbursement"[TiAb] OR "guideline"[TiAb] OR "mandate"[TiAb] OR "consensus*"[TiAb] OR "recommendat*"[TiAb] OR "standards"[TiAb] OR "best practice*"[TiAb]	1,210,048
3	**Clinical**	"clinical outcom*"[TiAb] OR "disease outcome"[TiAb] OR "clinical effectiveness"[TiAb] OR "clinician reported outcome"[TiAb] OR "physician reported outcome"[TiAb] OR "treatment outcome"[MeSH] OR "treatment outcome"[TiAb] OR "patient reported outcome measures"[MeSH] OR "Patient Reported Outcome"[TiAb] OR "PRO"[TiAb] OR "surrogate outcome"[TiAb] OR "framework"[TiAb] OR "clinical benefit"[TiAb] OR "treatment initiation"[TiAb] OR "treatment action"[TiAb] OR "medication adherence"[MeSH] OR "adherence"[TiAb] OR "efficacy"[TiAb] OR "health outcome"[TiAb] OR "patient outcome"[TiAb]	3,494,946
4	**Economic value**	"cost-effectiveness analysis"[MeSH] OR "Economics, Medical"[MeSH] OR "Budgets"[MeSH] OR "budget*"[tiab] OR "economic*"[tiab] OR "cost*"[tiab] OR "price*"[tiab] OR "value"[tiab] OR "models, economic"[MeSH] OR "economic model*"[tiab] OR "Cost-Benefit Analysis"[MeSH] OR "Cost Control"[MeSH] OR "Cost Savings"[MeSH] OR "Economic Competition"[MeSH] OR "Health Care Costs"[MeSH] OR "Health Expenditures"[MeSH] OR "ICER"[TiAb] OR "HCRU"[TiAb] OR "healthcare resource utilization"[TiAb] OR "healthcare resource utilisation"[TiAb] OR "healthcare utilisation"[TiAb] OR "healthcare utilisation"[TiAb] OR "healthcare cost"[TiAb] OR "cost utility"[TiAb] OR "utility"[TiAb] OR "health economics"[TiAb] OR "cost analysis"[TiAb] OR "cost-minimization"[TiAb] OR "cost-minimisation"[TiAb] OR "cost minimization"[TiAb] OR "cost minimisation"[TiAb] OR "budget impact"[TiAb] OR "economic evaluation"[TiAb] OR "costs and cost analysis"[MeSH]	2,942,476
5	**Additional**	"health equity"[MeSH] OR "health equity"[TiAb] OR "quality of life"[MeSH] OR "quality of life"[TiAb] OR "QoL"[TiAb] OR "quality-adjusted life years"[MeSH] OR "quality-adjusted life	604,285

		years"[TiAb] OR "QALY*"[TiAb] OR "disability-adjusted life years"[MeSH] OR "disability-adjusted life years"[TiAb] OR "DALY*"[TiAb] OR "life expectancy"[MeSH] OR "life expectancy"[TiAb] OR "patient experience"[TiAb]	
**6**	**Stakeholder**	"payer"[TiAb] OR "HTA"[TiAb] OR "health technology assessment"[TiAb] OR "insurer"[TiAb] OR "CMS"[TiAb] OR "medicare"[TiAb] OR "Medicaid"[TiAb] OR "national coverage determination"[TiAb] OR "local coverage determination"[TiAb] OR "USPSTF"[TiAb] OR "pharmacy & therapeutics committee"[TiAb] PR "pharmacy and therapeutics committee"[TiAb] OR "pharmacy and therapeutic committee"[TiAb] OR "P&T committee"[TiAb] OR "FDA"[TiAb] OR "NHS"[TiAb] OR "health system"[TiAb] OR "National Health Service"[TiAb] OR "NICE"[TiAb] OR "National Institute for Health and Care Excellence"[TiAb] OR "Gemeinsamer Bundesausschuss"[TiAb] OR "G-BA"[TiAb] OR "GKV-Spitzenverband"[TiAb] OR "G-KV"[TiAb] OR "LBM"[TiAb] OR "reimbursement"[TiAb]	236,200
**7**	**Combination**	#2 OR #3 OR #4 OR #5 OR #6	7,251,837
**8**	**Combination**	#1 AND #7	29,939
**9**	**With filters**	#9 AND y_5[Filter] AND fha[Filter] AND humans[Filter] AND english[Filter]	9,023
